# High thermal conductivity dielectric polymers show record high capacitive performance at high temperatures

**DOI:** 10.1093/nsr/nwad224

**Published:** 2023-09-01

**Authors:** Yang Shen, Ce-Wen Nan

**Affiliations:** School of Materials Science and Engineering, State Key Laboratory of New Ceramics and Fine Processing, Tsinghua University, China; School of Materials Science and Engineering, State Key Laboratory of New Ceramics and Fine Processing, Tsinghua University, China

Electrostatic capacitors are the most widely used components in modern electrical and electronic systems ranging from integrated circuits, electrical energy storage and pulsed power facilities. Polymers are the preferred dielectrics for electrostatic capacitors owing to their unique self-healing ability, high breakdown strength and ease of processing [1,2]. However, as the operating temperature increases, dielectric polymers exhibit substantially increased conduction loss, which leads to huge derating of electrical energy storage and even thermal runaway because of their low thermal conductivity (*κ* < 0.4 W m^−1^ K^−1^) [3]. Consequently, the high-temperature capacitive energy storage of dielectric polymers is inseparable from the cumbersome active cooling, which increases the extra volume, weight and energy consumption of integrated systems, and reduces their reliability and efficiency.

To efficiently dissipate heat energy generated by conduction loss, high thermal conductivity and low electrical conductivity are desirable for high-temperature dielectric polymers. However, high thermal conductivity and low electrical conductivity are a long-standing contradiction in the field of dielectric polymers. Recently, in a study led by Prof. Xingyi Huang from Shanghai Jiao Tong University [4], a ladderphane copolymer that integrates both high thermal conductivity and low electrical conductivity was first prepared by ring-opening metathesis polymerization (ROMP) (Fig. 1a). The copolymer self-assembles into highly ordered arrays by π–π stacking interactions (Fig. 1b), thus giving rise to an unprecedented intrinsic through-plane thermal conductivity of 1.96 ± 0.06 W m^−1^ K^−1^. Moreover, the copolymer demonstrates ∼40 times lower electrical conductivity when compared with the best available high-temperature dielectric polymer (i.e. polyetherimide), at elevated temperatures and high electric fields.

**Figure 1.**
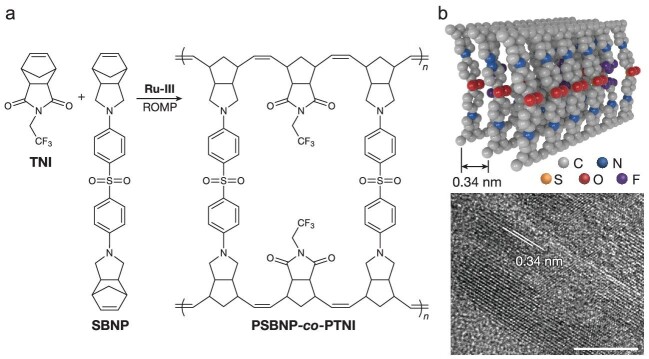
(a) Synthesis of ladderphane copolymer by ring-opening metathesis polymerization. (b) Schematic and transmission electron microscope image of the ordered self-assembled structures of the ladderphane copolymer. Scale bar, 10 nm. Adapted with permission from ref. [4]. Copyright 2023, Nature Publishing Group.

The capacitive performance showed that the copolymer outperforms the state-of-the-art high-temperature dielectric polymers and polymer composites. At a charge-discharge efficiency (*η*) ≥ 90%, the copolymer delivers a discharge energy density (*U*_d_) of 5.34 J cm^−3^ at 200°C, compared to *U*_d_ of 0.3 J cm^−3^ of polyetherimide, and *U*_d_ of 3.1 J cm^−3^ of the recently developed polyetherimide/molecular semiconductor composite. In continuous charge-discharge cyclic tests at 200°C, the generated Joule heat in the low thermal conductivity polyetherimide film (0.13 ± 0.03 W m^−1^ K^−1^) makes the temperature exceed 210^o^C. In contrast, the temperature of the copolymer film almost does not change during the charge-discharge cycle. Consequently, at 300 MV m^−1^, the PEI film can operate only up to 8741 cycles at 150°C. By contrast, *U*_d_ and *η* of the optimal copolymer are highly stable during 53 176 continuous charge-discharge cycles at 200°C. Moreover, the copolymer possesses an excellent self-healing ability due to its low C/(O+H) ratio (∼0.97), which can avoid catastrophic failure of capacitors due to accidental electrical breakdown [5].

This study provides a solution to the contradiction between high thermal conductivity and low electrical conductivity in dielectric polymers and represents a milestone that will push forward the development of dielectric polymers for high-temperature capacitive energy storage. In the future, research could focus on the synthesis technique for large-scale production of high-thermal-conductivity dielectric polymers.

## References

[1] Ren W , Yang M, Zhou L et al. Adv Mater 2022; 34: 2207421. 10.1002/adma.202207421 36210753

[2] Wen L , Chen J, Liang J et al. Natl Sci Rev 2017; 4: 20–3. 10.1093/nsr/nww041

[3] Chen J , Shen Z, Kang Q et al. Sci Bull 2022; 67: 609–18. 10.1016/j.scib.2021.10.011 36546122

[4] Chen J , Zhou Y, Huang XY et al. Nature 2023; 615: 62–6. 10.1038/s41586-022-05671-4 36859585

[5] Tan DQ. Adv Funct Mater 2020; 30: 1808567. 10.1002/adfm.201808567 PMC7357591 32684905

